# Scabronine G Methyl Ester Improves Memory-Related Behavior and Enhances Hippocampal Cell Proliferation and Long-Term Potentiation via the BDNF-CREB Pathway in Olfactory Bulbectomized Mice

**DOI:** 10.3389/fphar.2020.583291

**Published:** 2020-11-12

**Authors:** Osamu Nakagawasai, Jia-Rong Lin, Takayo Odaira, Kohei Takahashi, Wataru Nemoto, Shigeki Moriguchi, Yasushi Yabuki, Yu Kobayakawa, Kohji Fukunaga, Masahisa Nakada, Koichi Tan-No

**Affiliations:** ^1^ Department of Pharmacology, Faculty of Pharmaceutical Sciences, Tohoku Medical and Pharmaceutical University, Sendai, Japan; ^2^ Department of Pharmacology, School of Pharmacy, International University of Health and Welfare, Ohtawara, Japan; ^3^ Research Center for Pharmaceutical Development, Graduate School of Pharmaceutical Sciences, Tohoku University, Sendai, Japan; ^4^ Department of Pharmacology, Graduate School of Pharmaceutical Sciences, Tohoku University, Sendai, Japan; ^5^ Department of Genomic Neurology, Institute of Molecular Embryology and Genetics, Kumamoto University, Kumamoto, Japan; ^6^ Department of Chemistry and Biochemistry, Faculty of Science and Engineering, Waseda University, Tokyo, Japan

**Keywords:** long-term potentiation, memory, olfactory bulbectomy, scabronine G methyl ester, cell proliferation

## Abstract

A previous study reported that scabronine G methyl ester (SG-ME) potentially enhances the *in vitro* secretion of neurotrophic factors such as nerve growth factor via the protein kinase C (PKC)-ζ pathway. However, it remains unknown whether SG-ME can improve cognitive dysfunctions in olfactory bulbectomized (OBX) mice. To address this question, we evaluated SG-ME-treated and untreated OBX mice in a passive avoidance test. We also investigated potential effects of SG-ME on several parameters: cell proliferation and cAMP response element-binding protein (CREB) phosphorylation in the hippocampal dentate gyrus by immunohistochemistry, brain-derived neurotrophic factor (BDNF) levels in the hippocampus by Western blotting, *p*-CREB levels in the hippocampus by MapAnalyzer, and long-term potentiation (LTP) by electrophysiology. On the 14th day after surgery OBX mice showed altered passive avoidance and decreases in both cell proliferation and long-term potentiation in the hippocampus, while these changes were reversed by SG-ME (20 μg/mouse) 24 h after the treatment. The improvement in memory deficits was prevented when SG-ME was co-administeredwith either zeta inhibitory peptide (PKC-ζ inhibitor), anti-BDNF antibody, ANA-12 (TrkB antagonist), U0126 (MEK inhibitor), H-89 (PKA inhibitor), LY294002 (PI3K inhibitor) or KN-93 (CaMKII inhibitor). We found that SG-ME enhanced brain-derived neurotrophic factor and *p*-CREB levels in the hippocampus while *p*-CREB was localized in neurons, but not in astrocytes nor microglial cells. These findings revealed the potential of SG-ME in improving memory impairments by enhancing cell proliferation and LTP via activation of the BDNF/CREB signaling pathway in neurons.

## Introduction

An association between olfactory and psychic functions has been previously reported (Sohrabi et al., 2012). One of the evidence is that the olfactory bulb volume has been found to be smaller in Alzheimer’s disease (AD) and depressed patients than controls (Thomann et al., 2009; Negoias et al., 2010). In animals, olfactory bulbectomy (OBX) can lead to various behaviors including increased aggressive behavior (Cain, 1974), reduced sexual behavior (Larsson, 1971; Lumia et al., 1992), increased exploratory behavior (Sieck, 1972), cognitive dysfunction (Thorne et al., 1973; Yamamoto et al., 1997; Hozumi et al., 2003; Yabuki et al., 2017; Takahashi et al., 2018a), and depressive-like behaviors such as anhedonia (Sato et al., 2010) as well as prolonged immobility behavior (Takahashi et al., 2018b; Takahashi et al., 2018c). The integration of newborn neurons in the hippocampus influences animal behaviors, such as learning and memory (Snyder et al., 2005) and depressive-like behaviors (Snyder et al., 2011). A decline in adult neurogenesis is observed in AD, depression and schizophrenia patients (Hussaini et al., 2014). The OBX mice also showed diminished newborn neurons in the dentate gyrus (DG) of the hippocampus (Nakagawasai et al., 2016; Takahashi et al., 2018b). These abnormal behaviors and pathological changes in OBX rodents were rescued by antidementia drugs or chronic administration of antidepressant drugs (Saitoh et al., 2007; Yabuki et al., 2017; Takahashi et al., 2018b). Therefore, studies on the regulation of cell proliferation in the hippocampal DG of OBX mice could lead to therapeutic strategies against cognitive dysfunctions and depression.

Scabronines A-G were isolated from a bitter mushroom *Sarcodon scabrosus*. Scabronine G (SG), one member of the scabronine family, promoted the differentiation of PC-12 cells through the enhanced secretion of neurotrophic factors from 1321N1 human astrocytoma cells (Obara et al., 1999). The methyl ester form of SG (SG-ME) caused the enhancement of neurotrophic factor secretion from 1321N1 cells with a greater potency than the nonmethylester form (Obara et al., 2001). This beneficial effect of SG-ME was confirmed by Danishefsky’s lab who first achieved the total synthesis of SG-ME (Waters et al., 2005). In addition, cyrneines and glaucopine C, similar cyathane diterpenes isolated from *Sarcodon cyrneus*, promoted nerve growth factor (NGF) gene expression in 1321N1 cells, although these effects were weaker than SG-ME (Marcotullio et al., 2007). Preliminary experiments have also shown that the gene expression of brain-derived neurotrophic factor (BDNF), in addition to NGF, was significantly increased by SG-ME in 1321N1 cells. Evidence indicate that increased BDNF expression in the DG of the hippocampus relates to *in vivo* proliferation and differentiation of neuronal progenitor cells (Lee et al., 2002; Sairanen et al., 2005), induces long-term potentiation (LTP) (Gooney et al., 2004) and rescues cognitive dysfunctions (Ji et al., 2014). It has been reported that BDNF acts via tyrosine kinase B (TrkB) receptors and activates the mitogen-activated protein kinase (MAPK), protein kinase A (PKA), phosphoinositide 3-kinase (PI3K), Ca^2+^/calmodulin-dependent protein kinase II (CaMKII) pathway thereby promoting the phosphorylation of the transcription factor cAMP response element binding protein (CREB) (Hashimoto et al., 2004; Cunha et al., 2014; Manosso et al., 2015). The BDNF/CREB pathway plays a number of important roles in neurosurvival, synaptic plasticity and memory, and participate in the pathophysiology of both AD and depression. However, no studies have examined the pharmacological activity of SG-ME in rodents.

The aim of this study was to explore the effects of SG-ME on memory-related behavior impairment in the OBX mice as total synthesis of cyathane diterpenoid scabronine G was achieved (Kobayakawa and Nakada, 2013). In a final set of experiments aimed to identify the possible mechanisms involved in the regulation of cell proliferation and LTP in the hippocampus, we assessed the effect of SG-ME on BDNF/CREB signaling pathways in OBX mice.

## Experimental Procedures

All experiments were performed according to the Guide for Care and Use of Laboratory Animals from Tohoku Medical and Pharmaceutical University (Approval number: A14021cn, A15019cn, 16023cn, 17015cn) and according to the National Institutes of Health Guide for the Care and Use of Laboratory Animals. Efforts were made to minimize suffering and to reduce the number of animals used.

### Animals

Adult male ddY mice (Japan SLC Inc., Shizuoka, Japan) weighing 26–28 g were used in this study (total: n = 437; behavioral tests: n = 323; western blot analysis: n = 30; immunohistochemistry: n = 64; electrophysiology analysis: n = 20). The animals were housed together in polypropylene cages (13 cm height × 21 cm width × 31 cm length) with a stainless steel wire lid bedding material made of wood shavings (Soft chip, Japan SLC Inc., Shizuoka, Japan). Housing was under conditions of constant temperature (23 ± 1°C), humidity (55 ± 5%), with a 12∶12 h light–dark cycle (lights on 7∶00–19∶00 h); food and water were available ad libitum.

### Drugs and Treatments

The following compounds were used: SG-ME (provided by Drs. Kobayakawa and Nakada); Myristolated PKC Zeta, Pseudosubstrate (ZIP, Zeta-inhibitory peptide; AnaSpec, Inc, Kumamoto, Japan); Anti-BDNF antibody (Abcam; Cambridge, United Kingdom); 1,4-Diamino-2,3-dicyano-1,4-*bis*[2-aminophenylthio]butadiene (U0126; Tocris Bioscience, Minneapolis, United States); N-[2-[[(Hexahydro-2-oxo-1H-azepin-3-yl)amino]carbonyl]phenyl]benzo [b]thiophene-2-carboxamide (ANA-12; Tocris Bioscience, Minneapolis, United States);N-[2-(*p*-Bromocinnamylamino)ethyl]-5-isoquinolinesulfonamide dihydrochloride (H-89; Sigma-Aldrich, Missouri, United States); N-[2-[N-(4-Chlorocinnamyl)-N-methylamino-methyl]phenyl]-N-(2-hydroxyethyl)-4-methoxybenzenesulfonamide phosphate salt (KN-93; Calbiochem, Merck Millipore, Massachusetts, United States); and 2-(4-Morpholinyl)-8-phenyl-1(4H)-benzopyran-4-one hydrochloride (LY294002; Sigma-Aldrich). SG-ME was dissolved in ringer’s solution containing 10% dimethylsulfoxide (10% DMSO; Wako Pure Chemical Industries Ltd., Osaka, Japan). ZIP, Anti-BDNF antibody, H-89, and KN-93 were dissolved in ringer’s solution (Fuso Pharmaceutical Industries, Ltd., Osaka, Japan). ANA-12 was dissolved in saline (Fuso Pharmaceutical Industries). U0126 and LY294002 were dissolved in 10% DMSO. The dose for each drug used aside from SG-ME was taken from previous reports (Damaj, 2007; Migues et al., 2010; Schmid and Bohn, 2010; Qin et al., 2014; Bogard and Tavalin, 2015; Ma et al., 2016; Takahashi et al., 2017). All doses used in this study did not influence locomotor activity in mice (data not shown). 5 μL of all compounds except for ANA-12 (intraperitoneally; i.p.) were administrated intracerebroventricularly (i.c.v.) using a 50 μL Hamilton microsyringe attached to a disposable 27-G needle. All compounds except for SG-ME were administrated 24 h before the behavioral test.

### Surgical Operation

**FIGURE 1:**
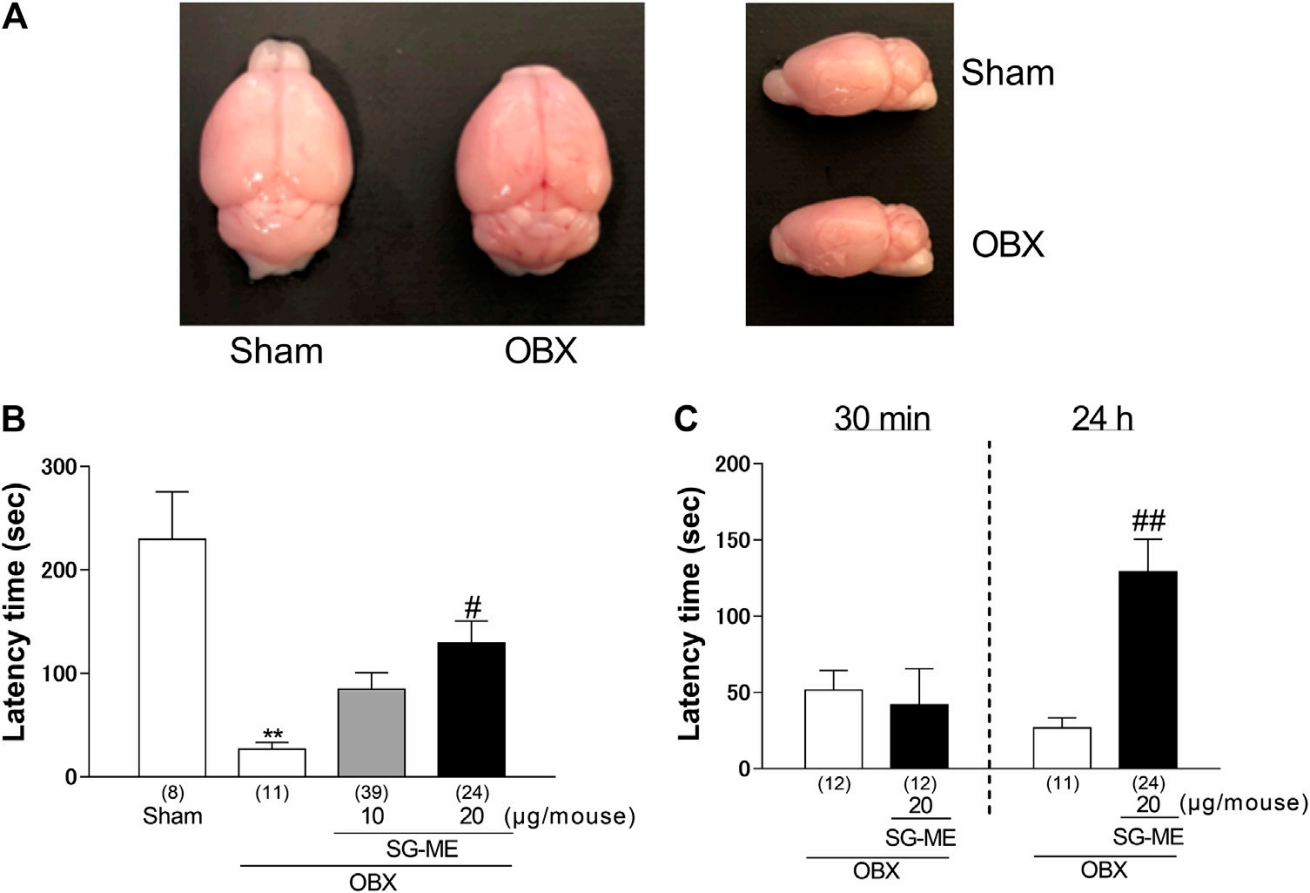
SG-ME prolonged the passive avoidance behavior in OBX mice. Representative images of whole brains from sham and OBX mice **(A)**. The latency times for the different treatment groups are shown for the retention trial in panel **(B)**. SG-ME or vehicle was injected i.c.v. 30 min or 24 h before the retention trial, respectively, and each mouse was used in a single trial **(C)**. Bars represent means ± SEM. **: *p* < 0.01 vs. vehicle-treated sham group. Numbers in parentheses indicate the number of animals in each group. #: *p* < 0.05 and ##: *p* < 0.01 vs. vehicle-treated OBX group.

Surgical OBX was performed as previously described (Hozumi et al., 2003; Odaira et al., 2019). The mice were anesthetized with sodium pentobarbital (50 mg/kg; Kyoritsu Seiyaku, Tokyo, Japan) and subsequently placed on a stereotaxic instrument. The head was incised and drilled on the sagittal midline, and the olfactory bulbs (OB)s were aspirated using a micropipette tip connected to a water vacuum pump. The mice were occluded in their anterior holes using a hemostatic sponge. All mice were euthanized at the end of the experiment and it was visually confirmed that two-thirds of the OB had been lesioned. Mice were excluded from the data if the lesion did not extend to more than two-thirds of the OB or if it extended to the cortex. Sham operations followed the same surgical procedure without the removal of the OB. The OBs of sham and OBX mice are shown in Figure 1A.

### Passive Avoidance Test

The passive avoidance test (PAT) was performed, as previously described (Hozumi et al., 2003). In brief, OBX was performed within 30 min after the training trial in which each mouse was placed in the illuminated compartment and the latency to enter the dark compartment was recorded. As soon as the mouse had left the lighted compartment and completely entered the dark compartment, an electric foot-shock (1 mA for 500 ms) was delivered to the feet through the floor grids. In the retention trial, each mouse for each time point was placed in the illuminated compartment and the latency to enter the dark compartment was once again recorded, allowing a maximum cut-off time of 300 s; no foot-shock was delivered in this case. We have previously reported that the impairment of memory-related behavior in OBX mice can be observed on the 14th day after the surgery or training trial (Hozumi et al., 2003). SG-ME was administered on the 14th day after the training trial which was followed by the retention trial either on the same day or on the 15th day to measure the duration of the drug effect 30 min and 24 h later, respectively. All measurements were performed between 10:00 and 17:00. In the following experiments we used samples from mice that had not undergone any behavioral testing.

### Western Blotting

We measured the expression of the BDNF protein in the hippocampi of mice on the 15th day after surgery. Mice (N = 30) were sacrificed by decapitation, brains were immediately removed and the dorsal hippocampi (left and right hippocampus for each mouse) dissected quickly using a mouse brain slicer (Muromachi Kikai, Tokyo, Japan). The brain atlas by Paxinos and Franklin (2001) was used as a reference to guide all dissections. Protein isolation and Western blots were performed as described previously (Nakagawasai et al., 2016; Takahashi et al., 2019a). After separation by electrophoresis, proteins were transferred from the gel onto polyvinylidene difluoride membranes using a semi-dry blotting apparatus (Bio-Rad Lab-oratories, Japan). The blots were blocked for 30 min with 5% skim-milk in Tris-buffered saline supplemented with 0.01% Tween-20 (TBST), then incubated in the presence of primary antibodies [diluted 1:200 for anti-BDNF or 1:1,000 for anti-β-actin (Cell Signaling Technology, Danvers, MA, United States) in TBST containing 5% skim-milk] overnight at 4°C. The blots were then washed several times followed by a 2 h incubation at room temperature in the presence of the secondary antibody (HRP-conjugated anti-rabbit IgG antibody diluted 1:5,000 with TBST containing 5% skim-milk). Immunoreactive proteins were revealed using an enhanced chemiluminescence (ECL) assay kit (GE Healthcare, Buckinghamshire, United Kingdom) with Hyper-film ECL. The density of the corresponding bands was analyzed using Image-J 1.43µ (National Institute of Health).

### Immunohistochemistry

#### Double Immunostaining

To assess hippocampal cell proliferation, mice (N = 21) were injected i. p. on the 14th day after surgery with 75 mg/kg of 5-Bromo-2′-deoxyuridine (BrdU; Nacalai Tesque, Inc.; Kyoto Japan) dissolved in saline every 2 h for a total of three times, after the administration of vehicle or SG-ME. Eighteen hours after the last BrdU injection, the animals were deeply anesthetized with sodium pentobarbital and brains were collected as described previously (Nakagawasai et al., 2016; Takahashi et al., 2019b). The brains were cut into 40 μm sections from bregma −1.40 mm to −2.00 mm using a cryostat (MICROM HM560, Microm Intstrument, Inc., California, United States). The distance between the sampled slides was 40 μm. The frozen sections were mounted on glass slides (Matsunami Glass, Osaka, Japan). Brain sections were treated with HCl (2 N) at 37°C for 30 min, followed by neutralization with sodium borate buffer (0.15 M) at room temperature (2 × 10 min). After three 5-min washes, the sections were incubated with phosphate-buffered saline (PBS) containing 1% normal goat serum and 0.3% Triton X-100 (PBSGT) at room temperature for 2 h. After this blocking and permeabilization step, the sections were incubated overnight at 4°C with the following primary antibodies: anti-*p*-CREB rabbit antibody (1:500; Wako Pure Chemical Industries), Anti-*p*-CREB mouse antibody (1:50; Enzo Life Sciences, Inc., New York, United States), anti-doublecortin (DCX) mouse antibody (1:50; Santa Cruz Biotech, Santa Cruz, United States) for immature neurons, anti-neuronal nuclei (NeuN) mouse antibody (1:500; Millipore Corporation, New York, United States) for mature neurons, anti-glial fibrillary acidic protein (GFAP) mouse antibody (1:200; MILLIPORE Corporation) for astrocytes, anti-ionized calcium binding adaptor molecule 1 (Iba1) rabbit antibody (1:100; Wako Pure Chemical Industries) for microglia, and anti-BrdU rat monoclonal antibody (1:100; AbD Serotec, Kidlington, United Kingdom). On the next day, the sections were washed again then incubated shielded from light at room temperature for 2 h, with the appropriate following secondary antibodies: Alexa Fluor 488 goat anti-mouse IgG (1:200), Alexa Fluor 568 goat anti-rabbit IgG (1:200), Alexa Fluor 488 goat anti-rabbit IgG (1:200), Alexa Fluor 568 goat anti-mouse IgG (1:200), Alexa Fluor 568 goat anti-rat IgG (1:200) (all from Molecular probes, Waltham, United States) in PBSGT. The sections were washed and mounted on slides with Dako fluorescent mounting medium (Dako, Carpinteria, United States). Colocalization of different antigens was performed using combinations of different primary and secondary antibodies. Immunofluorescent images were analyzed with a confocal laser-scanning microscope (A1Rsi: Nikon, Tokyo, Japan). Eight sections per mouse were used, and two images (left and right hemisphere, 640 μm × 640 μm) of the DG region of the hippocampus were obtained from each section. A blinded and independent observer counted the number of BrdU^+^/DCX^+^ cells in the DG for the analysis of cell proliferation. A mean number of eight images were analyzed for each mouse, and each group contained 5–6 mice.

#### Immunofluorescence Intensity of *p*-CREB in the Hippocampus

On the 15th day after surgery, mice (N = 43) were deeply anesthetized with sodium pentobarbital then perfused intracardially with 15 ml of cold PBS (4°C) followed by 45 ml of 4% PFA plus 25% glutaraldehyde in 0.1 M PBS. The brains were post-fixed in 4% PFA-0.1 M PBS for 1 h at 4°C, followed by immersion in 20% sucrose-0.1 M PBS for 48 h. The brains were cut into 20 μm-thick sections from bregma –1.40 to –2.00 mm using a cryostat. The distance between the sampled slides was 40 μm. The sections were washed twice for 30 min eachthen incubated in PBSGT at 4°C for 30 min followed by two washes for 15 min each. The sections were then incubated overnight at 4°C with anti-*p*-CREB rabbit antibody (1:500; Wako Pure Chemical Industries). On the following day, the sections were washed four times at room temperature for 30 min each, followed by an incubation with FITC-goat anti-rabbit IgG (1:200; Invitrogen, California, United States) for 3 h protected from light. Finally, the sections were washed four times 60 min at room temperature. The stained sections were coverslipped with Dako fluorescent mounting medium (Dako). The distribution of the *p*-CREB immunofluorescence intensity was quantitatively analyzed using a MapAnalyzer (Yamato Scientific Co., Ltd., Tokyo, Japan), as described previously (Takahashi et al., 2016; Sakuma et al., 2020). The values for immunohistochemical fluorescence intensity obtained for the various regions were measured relative to 1 mM quinine sulfate used as a standard (Sutoo et al., 1998). A mean number of ten images were analyzed for each mouse, and each group contained 10–12 mice.

### Electrophysiology Analysis

Hippocampal slices were prepared from mice (N = 20) brains 15 days following surgery, as described previously (Moriguchi et al., 2008). Transverse hippocampal slices (400 μm thick) prepared using a vibratome (Microslicer DTK-1000) were incubated for 2 h in continuously oxygenized (95% O_2_, 5% CO_2_) artificial cerebrospinal fluid (A-CSF) at 28°C. After a 2 h recovery period, slices were transferred to an interface recording chamber and perfused at a flow rate of 2 ml/min with A-CSF warmed to 34°C. Field excitatory post-synaptic potentials (fEPSPs) were evoked by a 0.05 Hz test stimulus through a bipolar stimulating electrode placed on the Schaffer collateral/commissural pathway and recorded from the stratum radiatum of cornu ammonis (CA) one region using a glass electrode filled with 3 M NaCl.

### Statistical Analysis

Results of experiments are expressed as mean ± SEM. Normality and homoscedasticity assumptions were verified prior to the use of any parametric tests (Shapiro-Wilk normality test and equality of variances F-test). The statistical significance of differences was determined by the Student’s t-test for two-group comparisons. The significance of differences was determined by one- or two-way analysis of variance (ANOVA), followed by Tukey-kramer test for multiple group comparisons except for LTP which was analyzed using Sheffe’s test. The criterion of significance was set at *p* < 0.05.

## Results

### Effect of SG-ME on the Passive Avoidance Behavior of OBX Mice

**FIGURE 2:**
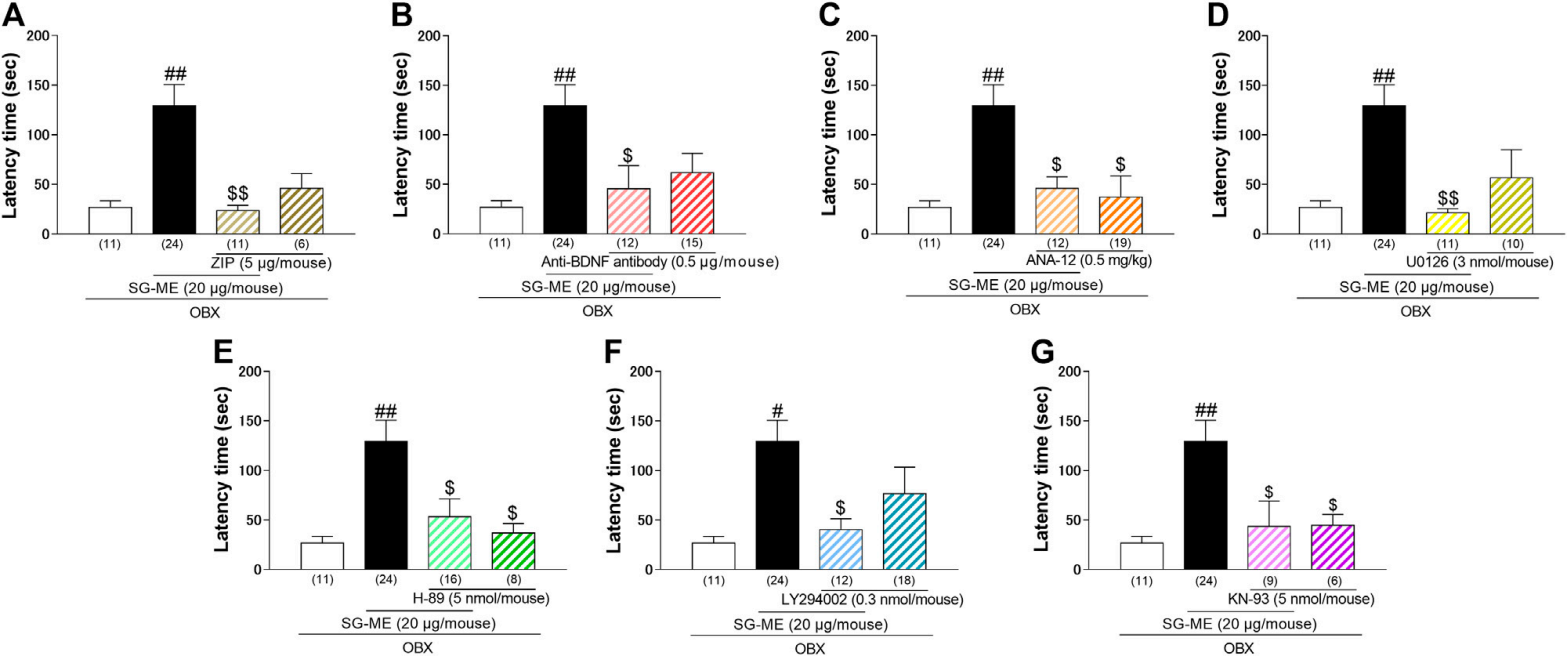
Effect of ZIP **(A)**, anti-BDNF antibody **(B)**, ANA-12 **(C)**, U0126 **(D)**, H-89 **(E)**, LY294002 **(F)** or KN-93 **(G)** on the amelioration of cognitive dysfunctions induced by SG-ME in OBX mice. Bars represent means ± SEM. Numbers in parentheses indicate the number of animals in each group. #: *p* < 0.05 and ##: *p* < 0.01 vs. vehicle-treated OBX group. $: *p* < 0.05 and $$: *p* < 0.01 vs. SG-ME (20 μg/mouse)-treated OBX group.

We evaluated the antidementia effect of SG-ME using the passive avoidance test. As reported previously with OBX mice, the post hoc test revealed the impairment in the avoidance behavior of vehicle-treated OBX mice compared to vehicle-treated sham mice (*p* = 0.0002). The i.c.v. administration of SG-ME (20 μg/mouse) in OBX mice significantly increased the latency time compared to vehicle-treated OBX mice (*p* = 0.0271) [One-way ANOVA; F (3, 78) = 7.647, *p* = 0.0002, Figure 1B]. A two-way ANOVA revealed a significant interaction between treatment and time [F (1, 55) = 6.573, *p* = 0.0131], with a significant main effect for treatment [F (1, 55) = 4.518, *p* = 0.0381]. The effect of SG-ME (20 μg/mouse) was observed 24 h but not 30 min after the injection (*p* = 0.0047) [Figure 1C]. These results suggest that SG-ME improves the memory-related behavior impairment in OBX mice. The increased latency time in SG-ME treated OBX mice was markedly inhibited by cotreatment of SG-ME with either ZIP (*p* = 0.0017) [One-way ANOVA; F (3, 48) = 7.843, *p* = 0.0002, Figure 2A], anti-BDNF antibody (*p* = 0.0319) [One-way ANOVA; F (3, 58) = 5.145, *p* = 0.0032, Figure 2B], ANA-12 (*p* = 0.0175) [One-way ANOVA; F (3, 52) = 6.717, *p* = 0.0006, Figure 2C], U-0126 (*p* = 0.0027) [One-way ANOVA; F (3, 52) = 6.896, *p* = 0.0005, Figure 2D], H-89 (*p* = 0.0206) [One-way ANOVA; F (3, 55) = 6.236, *p* = 0.0010, Figure 2E], LY294002 (*p* = 0.0335) [One-way ANOVA; F (3, 61) = 4.486, *p* = 0.0066, Figure 2F] or KN-93 (*p* = 0.0404) [One-way ANOVA; F (3, 49) = 5.892, *p* = 0.0016, Figure 2G].

### Changes of Hippocampal BDNF Immunocontent in SG-ME Treated OBX Mice

**FIGURE 3:**
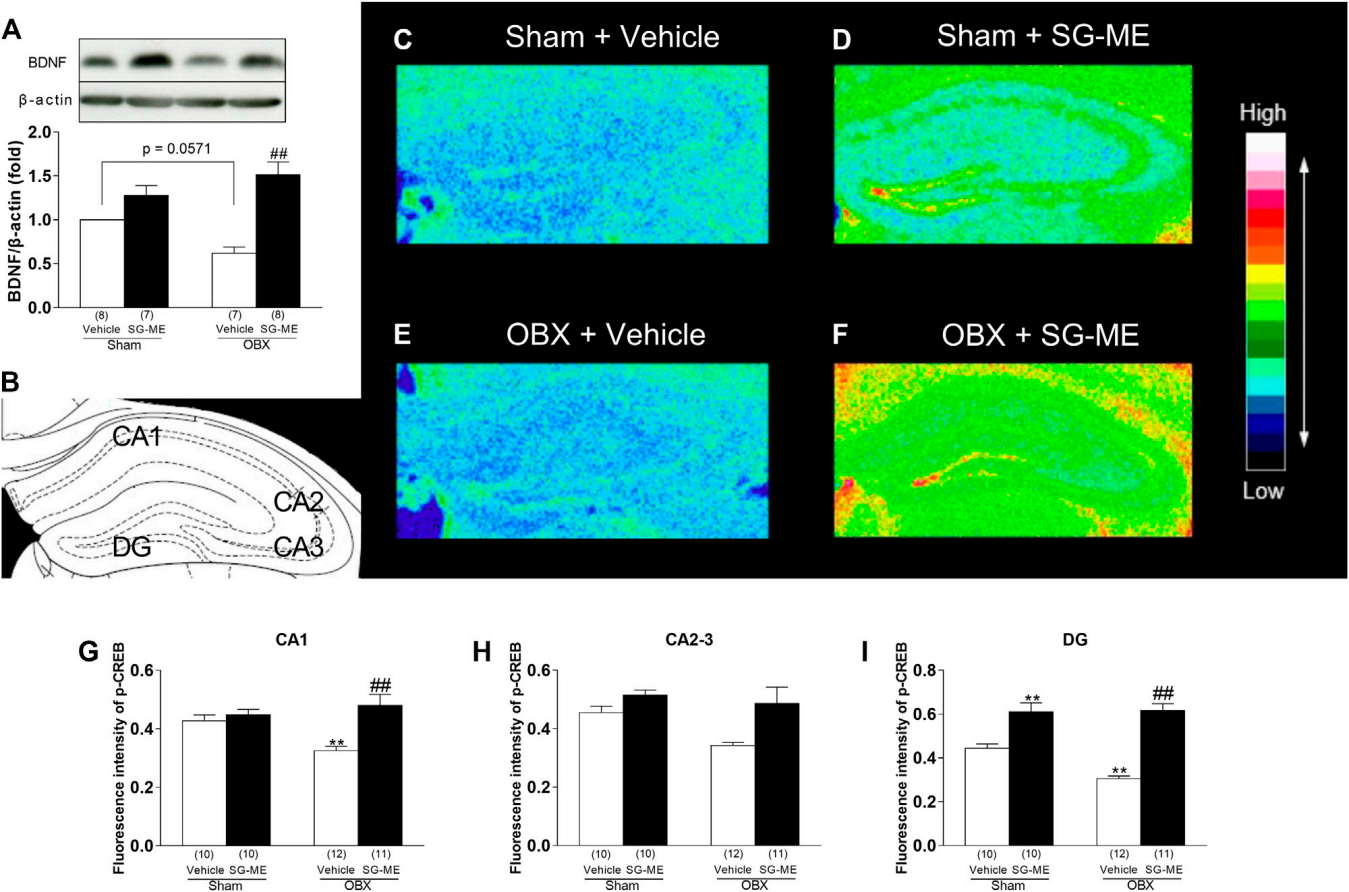
Increased BDNF and *p*-CREB levels in the hippocampus after SG-ME administration. Representative immunoblots probed with antibodies against hippocampal BDNF and *β*-actin, as indicated. Quantification of values for BDNF normalized with *β*-actin levels in the hippocampus **(A)**. Distribution of the immunohistochemical fluorescence intensity for *p*-CREB in the mouse hippocampus: diagram showing the hippocampal CA1, CA2, CA3, and DG areas **(B)**; pseudocolor images for **(C)** Sham + vehicle; **(D)** Sham + SG-ME; **(E)** OBX + vehicle; **(F)** OBX + SG-ME with corresponding intensity values (arbitrary units) for *p*-CREB in CA1 **(G)**, CA2-3 **(H)**, and DG **(I)**. Bars represent means ± SEM. Numbers in parentheses indicate the number of animals in each group. *: *p* < 0.05 and **: *p* < 0.01 vs. vehicle-treated sham group. #: *p* < 0.05 and ##: *p* < 0.01 vs. vehicle-treated OBX group.

The administration of SG-ME to OBX mice significantly increased BDNF levels in the hippocampus compared with vehicle-treated OBX mice (*p* < 0.0001). The vehicle-treated OBX group showed a tendency toward a decrease in BDNF expression compared to vehicle-treated sham group (*p* = 0.0571) [Figure 3A]. A two-way ANOVA revealed a significant interaction between group and treatment [F (1, 26) = 9.415, *p* = 0.0050], with a significant main effect for treatment [F (1, 26) = 33.91, *p* < 0.0001].

### Quantitative Immunofluorescence Analysis of *p*-CREB and Its Distribution in the Hippocampus of SG-ME- Treated OBX Mice

The fluorescence intensity for *p*-CREB in the hippocampal CA1 and DG areas of the OBX group was significantly decreased compared to the sham group (CA1: *p* = 0.0263; DG: *p* = 0.0044), whereas these areas showed increased levels after treatment with SG-ME (CA1: *p* = 0.0003; DG: *p* < 0.0001) (Figures 3G,I). A two-way ANOVA revealed an interaction between group and treatment in CA1 [F (1, 39) = 7.172, *p* = 0.0108] and DG [F (1, 39) = 6.943, *p* = 0.0120]. However, the two-way ANOVA did not reveal any group × treatment interaction in CA2-3 [F (1, 39) = 1.724, *p* = 0.1968, Figure 3H]. Next, we performed dual immunofluorescence stainings for *p*-CREB and different markers, such as NeuN (neuronal marker), GFAP (astrocytic marker) or Iba1 (microglial marker) in the hippocampal DG of OBX mice, to determine the cell types activated by SG-ME. Following SG-ME administration, *p*-CREB labeling was found in NeuN-positive cells. On the other hand, *p*-CREB-labeled cells were not observed in GFAP- and Iba1-positive cells.

### Influence of SG-ME on the Reduced Hippocampal Cell Proliferation in OBX Mice

The number of double-labeled BrdU^+^/DCX^+^ cells in the vehicle-treated OBX group was significantly decreased compared to the vehicle-treated sham group (*p* = 0.0323) [Figures 5A,B]. Conversely, a significant increase in the number of these cells was found in the SG-ME-treated OBX mice compared with the vehicle-treated OBX group (*p* < 0.0001) [Figures 5A,B]. A two-way ANOVA revealed an interaction between group and treatment on the number of BrdU-positive cells expressing DCX [F (1, 17) = 15.37, *p* = 0.0011].

### Influence of SG-ME on LTP Impairment in the Hippocampal CA1 Region of OBX Mice

We analyzed LTP induced by high-frequency stimulation (HFS; 100 Hz, two trains) of collateral/commissural pathways, using hippocampal slices obtained from sham and OBX mice treated with vehicle or SG-ME. A two-way ANOVA revealed an interaction between group and treatment 60 min after HFS [F (1, 16) = 5.603, *p* = 0.0309]. HFS produced a stable and long-lasting increase in fEPSPs in vehicle-treated sham mice, whereas hippocampal LTP was significantly reduced in vehicle-treated OBX mice compared to sham mice 60 min after HFS (*p* = 0.0398) [Figure 6D]. SG-ME administration (20 μg/mouse) significantly improved LTP in the hippocampal CA1 of the OBX mice 60 min after HFS (*p* = 0.0327) [Figure 6D]. However, a two-way ANOVA revealed no interactions with time-dependent changes [F (207, 1,120) = 0.6397, *p* > 0.9999, Figure 6B] or 1 min after HFS [F (1, 39) = 1.724, *p* = 0.1968, Figure 6C].

## Discussion

In this study, we examined the effects and underlying mechanisms of SG-ME on the memory-related behavioral impairment in OBX mice.

Our laboratory and other researchers reported that OBX rodents represent not only a model for depressive-like behaviors (Sato et al., 2010; Takahashi et al., 2018b; Takahashi et al., 2018c) but also for cognitive dysfunctions (Yamamoto et al., 1997; Slotkin et al., 1999; Hozumi et al., 2003). Our previous studies have shown that OBX induces memory impairment in the PAT which has the ability to evaluate long-term memory (Hozumi et al., 2003; Takahashi et al., 2017, Takahashi et al., 2018a). The present study revealed that SG-ME improves the memory impairment in OBX mice 24 h but not 30 min after its i.c.v. administration at a dose of 20 μg/mouse [Figures 1B,C], while no effect was observed in sham mice treated with SG-ME (Supplementary Figure S1). These results suggest that SG-ME has an anti-dementia effect in OBX mice.

In previous study, Obara et al. demonstrated that SG-ME enhanced neurotrophic factors biosynthesis, which plays critical roles in memory and cognitive functions, via PKC-ζ and possibly nuclear factor-kappa B (NF-κB) (Obara et al., 2001). Moreover, we reported that the cell proliferation promoted via the PKC-ζ/NF-κB/BDNF/TrkB/CREB signaling pathway in hippocampal precursor neurons occurred concomitantly with an anti-depressant effect in OBX mice (Odaira et al., 2019). The present PAT showed that the PKC-ζ inhibitor zeta inhibitory peptide (ZIP) attenuated the SG-ME-induced improvement in memory deficits in OBX mice [Figure 2A]. Taken together, these results suggest that the memory improvement induced by SG-ME may be related to the activation of the PKC-ζ pathway.

SG-ME enhanced the secretion of the neurotrophic factor NGF from 1321N1 human astrocytoma cells which was accompanied by the activation of PKC-ζ (Obara et al., 2001). Our preliminary experiment indicated that the expression of BDNF was also induced by the SG-ME treatment (data not shown). In addition, it has been suggested that a decrease in BDNF, but not NGF nor NT-3 expression levels occurs in the brain of AD patients, particularly in the hippocampus and cerebral cortex (Phillips et al., 1991). Therefore, we examined and established the potential role of BDNF in the anti-dementia effect of SG-ME by demonstrating that the effect was lost when an antibody against BDNF was co-administered [Figure 2B]. Furthermore, our Western blotting experiments showed that the expression of BDNF was increased in the hippocampus of OBX mice following SG-ME treatment [Figure 3A]. Since BDNF specifically binds to the TrkB receptor, we hypothesized that the anti-dementia effect of SG-ME may be blocked by ANA-12, a selective TrkB receptor antagonist. Indeed, we found that the co-administration of ANA-12 and SG-ME in OBX mice markedly inhibited the improvement in their memory deficits otherwise induced by SG-ME alone. Likewise, Jiang et al. reported that the anti-depressant effects as well as the enhancement of cell proliferation induced by ginsenoside Rg1 were blocked by the co-administration of K252a, a potent inhibitor of TrkB receptor (Jiang et al., 2012). Thus, we believe that BDNF is involved in the SG-ME-induced memory improvement we observed.

BDNF signals are transduced to the MAPK, PI3K, and/or CaMKII cascades via TrkB (Yamada and Nabeshima, 2003). The MEK/ERK/CREB pathway is one of the three currently known MAPK pathways including the MEK/ERK/CREB pathway communicate through phosphorylation of neighboring proteins. Specifically, it receives messages from receptors and sequentially activates Ras, RAf, MEK/ERK, RSK, leading to the phosphorylation of CREB and ultimately to the promotion of cell proliferation or neurogenesis (McCubrey et al., 2007; Deinhardt and Chao, 2014). The present study showed that the MEK inhibitor U0126 significantly inhibited the memory improvement induced by SG-ME in OBX mice [Figure 2D]. The ERK-signaling pathway not only regulates long-term synaptic plasticity and hippocampal-dependent learning, but it is also involved in the structural remodeling of excitatory spine synapses triggered by BDNF (Alonso et al., 2004). Thus, the effects of SG-ME may involve the ERK signaling pathway. Next, we used the selective PKA inhibitor H-89 to examine whether the PKA-signaling pathway was associated with the effect of SG-ME. Our data shows that the SG-ME-induced memory improvement was significantly suppressed by the co-administration of H-89 [Figure 2E]. It has been reported that the infusion of H-89 in the hippocampus impaired spatial memory retention (Sharifzadeh et al., 2006). Moreover, a previous study has suggested that synaptic protein levels and dendritic outgrowth in hippocampal neurons are strongly related to the PI3K- and CaMKII- signaling pathways (Seo et al., 2014). We used LY294002, a PI3K inhibitor, and KN-93, a CaMKII inhibitor, to investigate whether these signaling pathways are also related to the effects of SG-ME. Indeed, the SG-ME-induced memory improvement was inhibited by either LY294002 [Figure 2F] or KN-93 [Figure 2G]. In summary, the effects of SG-ME observed in the hippocampus of OBX mice appear to involve the cAMP/PKA-, PI3K- and CaMKII-signaling pathways.

**FIGURE 4:**
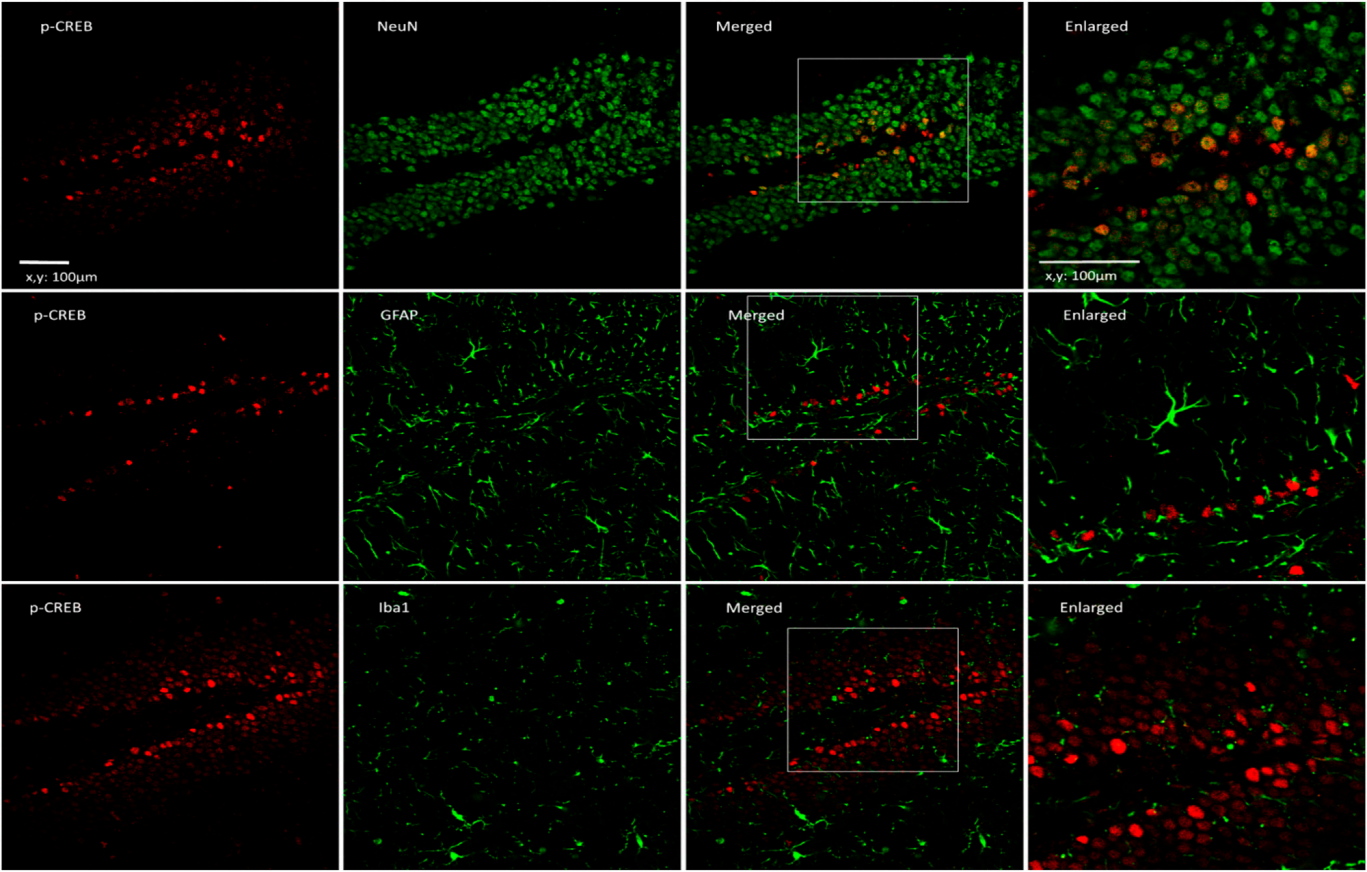
p-CREB is expressed in neurons of the hippocampus in OBX mice treated with SG-ME. Microscopy images of *p*-CREB (red) together with NeuN, GFAP or Iba1 (green) immunostaining in the DG region of the hippocampus (Scalebar: 100 μm). The boxed areas are shown with higher magnification (Scalebar: 100 μm).

CREB plays an important role in transducing the signal from BDNF to transcription factors and mediating neuronal plasticity. The activation of CREB has been shown to be involved in learning and memory formation (Kida, 2012). Conversely, anti-BDNF antibody administrated to rats impaired both short-term memory and long-term memories while decreasing the phosphorylation state of CREB (Alonso et al., 2005). *p*-CREB is essential for memory consolidation and storage in the hippocampus (O’Connell et al., 2000), and increases in the hippocampus following the chronic administration of anti-dementia (Nibuya et al., 1996). It has been determined that the activation of BDNF/CREB signaling may enhance newborn neurons and memory function (Walton et al., 1999). These findings suggest that increasing CREB activity may prove to be beneficial against dementia. The present study showed that while hippocampal *p*-CREB levels were decreased in OBX compared to sham mice (Figure 3), they increased following SG-ME in concomitance with *p*-CREB fluorescence intensity in the DG which localized to neurons, but not to astrocytes nor to microglia (Figure 4). Our group as well as others previously reported that BDNF and *p*-CREB levels were decreased in the hippocampus of OBX mice (Thakare et al., 2017; Takahashi et al., 2018b; Xu et al., 2018; Odaira et al., 2019; Takahashi et al., 2020), whereas the expression of BDNF in the hippocampus of vehicle-treated OBX in the present study mice also showed a tendency toward a decrease compared to the vehicle-treated sham group (*p* = 0.0571). Taken together, the above data indicate that cell proliferation in the hippocampal DG is affected by changes in BDNF and *p*-CREB levels and suggest that the activation of BDNF/CREB signaling pathways could be responsible for the effects of SG-ME.

**FIGURE 5:**
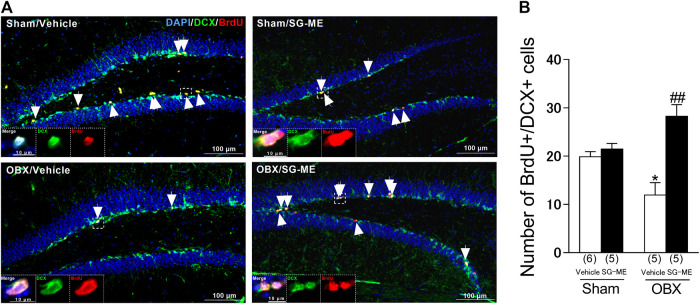
Influence of SG-ME on hippocampal cell proliferation in OBX mice. Microscopy images of BrdU (red) and DCX (green) immunostaining and DAPI (blue) in the DG region of the hippocampus **(A)**. Scalebar: 100 μm. Arrows indicate BrdU/DCX double positive cells. The boxed areas are shown at higher magnification. Quantitative analysis of the number of BrdU/DCX double positive cells in sham and OBX mice following vehicle or SG-ME administration **(B)**. Bars represent means ± SEM. Numbers in parentheses indicate the number of animals in each group. *: *p* < 0.05 vs. vehicle-treated sham group. ##: *p* < 0.01 vs. vehicle-treated OBX group.

The improvement in passive avoidance memory was accompanied by the enhancement of cell proliferation in the hippocampal DG, suggesting that an increase in newborn neurons was involved in retention and recall memory (Oh et al., 2006; Yang et al., 2008; Kim and Park, 2017; Takahashi et al., 2017). In the present study, OBX mice showed a significant decrease in cell proliferation in the DG compared to the sham group which is consistent with our previous study (Nakagawasai et al., 2016; Takahashi et al., 2017, Takahashi et al., 2018b), while this change was reversed by the SG-ME treatment [Figure 5B]. A reduction in newborn neurons in the hippocampus of patients with depression (DeCarolis and Eisch, 2010) and AD (Lazarov and Marr, 2010), two conditions known for decreased neuroplasticity, has also been reported. Inversely, increasing adult-born neurons was shown to be associated with an improvement in spatial memory-related behavior (Oh et al., 2013). Thus, it may be hypothesized that the SG-ME treatment improved the memory-related behavioral impairment in the OBX model by enhancing hippocampal cell proliferation.

**FIGURE 6:**
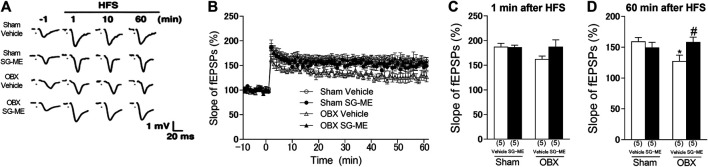
SG-ME improves LTP in the hippocampal CA1 region. Representative fEPSPs recordings from the CA1 region in sham + vehicle, sham + SG-ME, OBX + vehicle and OBX + SG-ME **(A)**. Changes in slope of fEPSPs following HFS recorded in the CA1 region was attenuated in OBX mice compared with that in sham mice, while SG-ME significantly improved LTP in OBX mice **(B)**. Bar graph representations of changes in slope of fEPSPs in sham + vehicle, sham + SG-ME, OBX + vehicle and OBX + SG-ME 1 **(C)** or 60 min **(D)** following HFS. Bars represent means ± SEM. Numbers in parentheses indicate the number of animals in each group. *: *p* < 0.05 vs. vehicle-treated sham group. #: *p* < 0.05 vs. vehicle-treated OBX group.

Hippocampal LTP is the molecular mechanism underlying cognitive function (Bliss and Collingridge, 1993). Moreover, LTP enhances the generation of newborn neurons in the hippocampus of DG (Takeuchi et al., 2018). BDNF, PKA, ERK1/2 and CaMKII are thought to play crucial roles in the induction of hippocampal CA1 LTP or its maintenance through phosphorylation of CREB (Fukunaga et al., 1993; Kiprianova et al., 1999; Xia and Storm 2005). Here, we observed that the LTP in the hippocampal CA1 area of vehicle treated OBX mice was significantly reduced compared to sham mice, which is consistent with our previous study (Moriguchi et al., 2009), but was improved by the SG-ME treatment [Figure 6D]. Likewise, *p*-CREB levels in the hippocampal CA1 area of OBX mice was significantly decreased compared to sham mice, while they improved after the SG-ME treatment [Figure 3G]. These results suggest that the enhancement of LTP by SG-ME in the hippocampal CA1 area may be related to the improvement of the memory-related behavioral impairment in the OBX model via the activation of *p*-CREB.

In the present study, we have evaluated memory function using the passive avoidance test only. We comprehend that this single method to assess memory limits our interpretation in the context of dementia. Hence, we plan to perform additional memory-related behavior tests such as those related to working or spatial memory in a future study.

**FIGURE 7:**
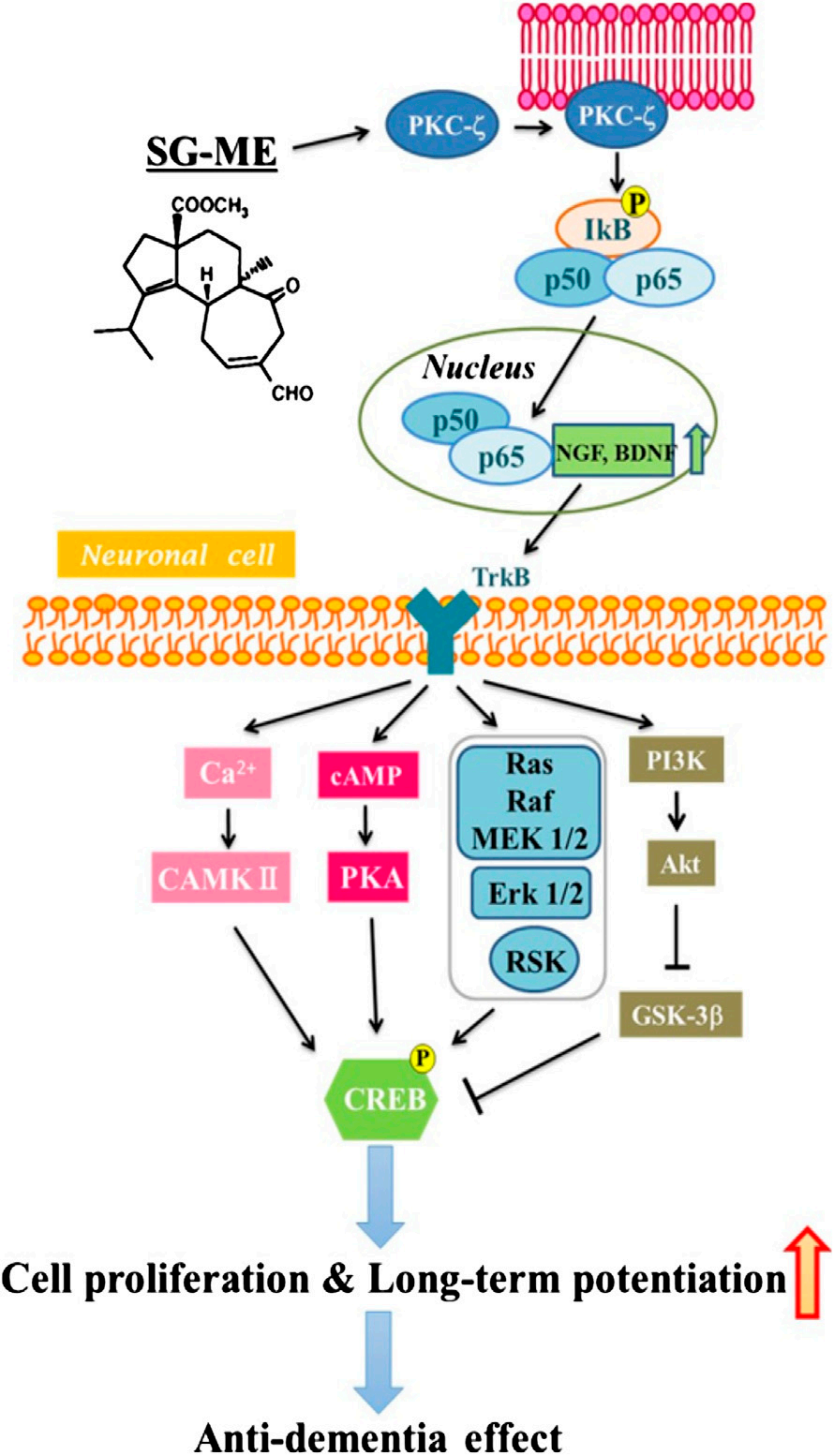
Schematic summary of the pathways through which SG-ME may signal and produce an anti-dementia affect.

In conclusion, the present study indicates that the potential anti-dementia effect of SG-ME would involve the enhancement of cell proliferation and LTP resulting from the activation of BDNF/CREB signaling pathways in neurons (Figure 7). Our results suggest that SG-ME could represent a candidate drug to consider in the development of a treatment against dementia.

## Data Availability Statement

The datasets presented in this study can be found in online repositories. The names of the repository/repositories and accession number(s) can be found in the article/supplementary material.

## Ethics Statement

The animal study was reviewed and approved by Care and Use of Laboratory Animals from Tohoku Medical and Pharmaceutical University. Written informed consent was obtained from the owners for the participation of their animals in this study.

## Author Contributions

ON: Validation, Conceptualization, Writing - original draft, Writing - review and editing, Project administration, Funding acquisition; JL: Investigation, Formal analysis, Writing - original draft; TO: Investigation, Formal analysis; KT: Formal analysis, Writing - original draft, Writing - review and editing; WN: Methodology, Writing–review and editing; SM: Investigation, Formal analysis; YY: Investigation; YK: Investigation; KF: Writing–review and editing; MN: Project administration, Writing–review and editing; KT: Conceptualization, Supervision, Funding acquisition.

## Funding

This study was supported in part by the Grants-in-Aid for Scientific Research (Grant number 18K06687 and 19K23808) and Matching Fund Subsidy for Private University from the Ministry of Education, Culture, Sports, Science and Technology of Japan (grant number S1511001 L).

## Conflict of Interest

The authors declare that the research was conducted in the absence of any commercial or financial relationships that could be construed as a potential conflict of interest.
