# Supplementary figures and images for: Scabronine G Methyl Ester Improves Memory-Related Behavior and Enhances Hippocampal Cell Proliferation and Long-Term Potentiation via the BDNF-CREB Pathway in Olfactory Bulbectomized Mice
Source: Front Pharmacol. 2020 Nov 12;11:583291. doi: 10.3389/fphar.2020.583291 (PMC7689418; doi:10.3389/fphar.2020.583291)

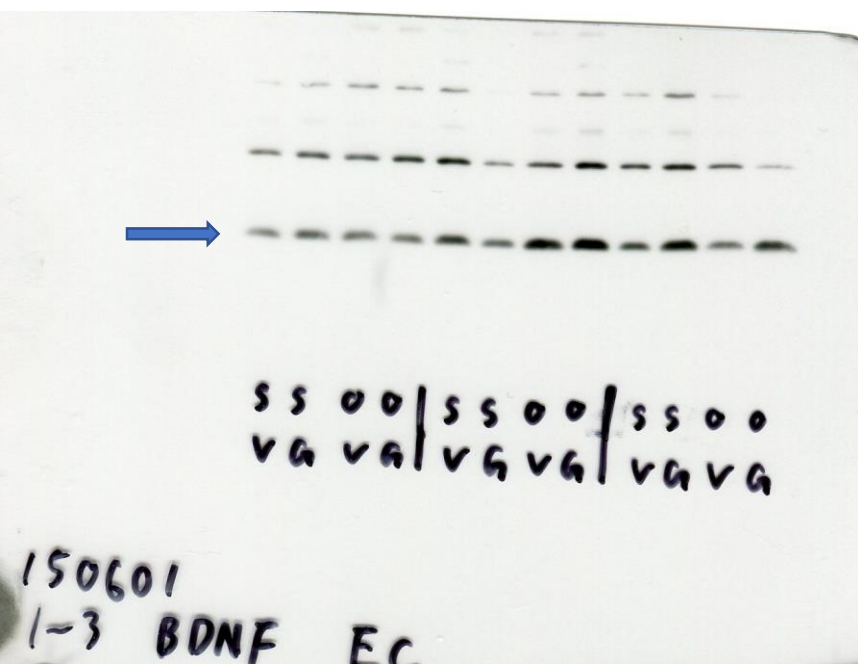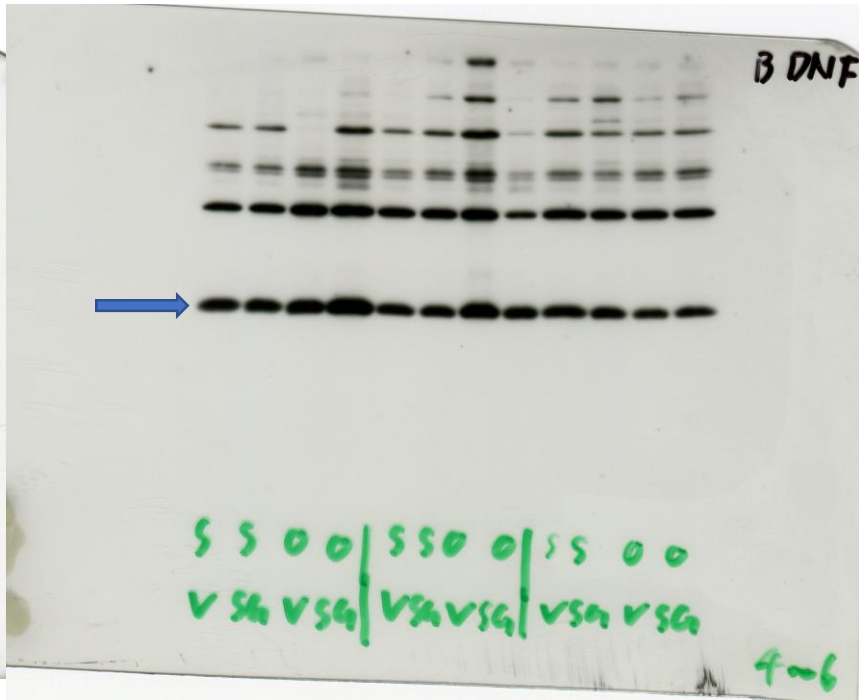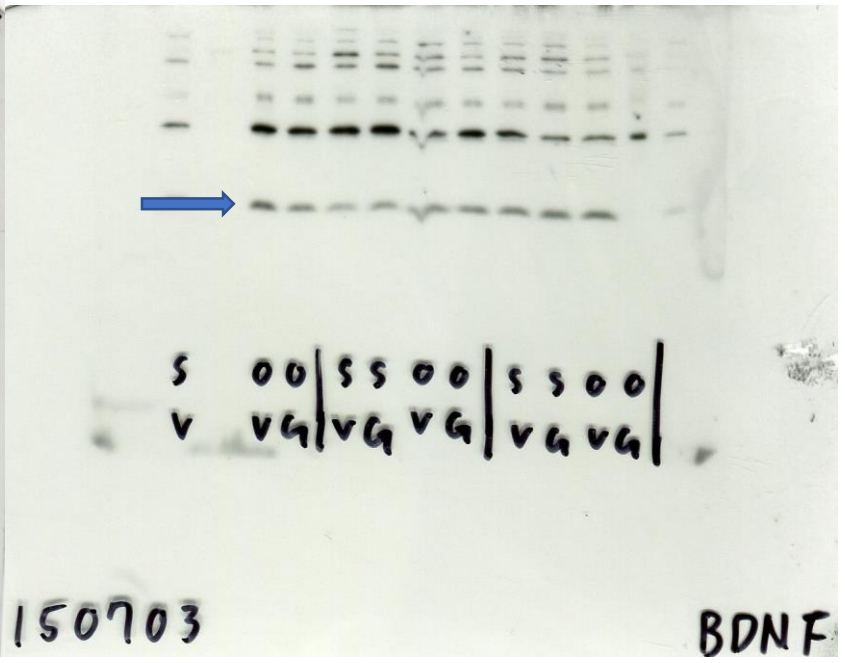

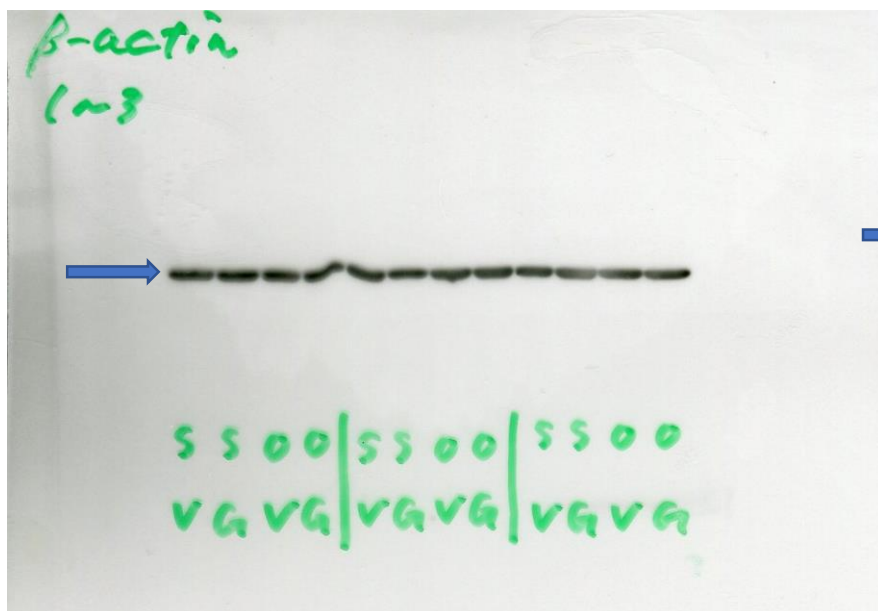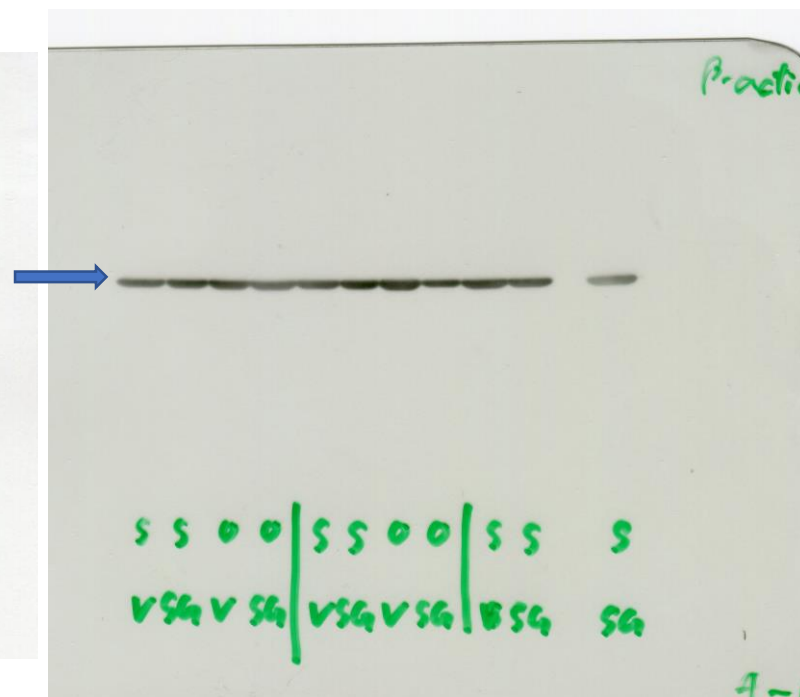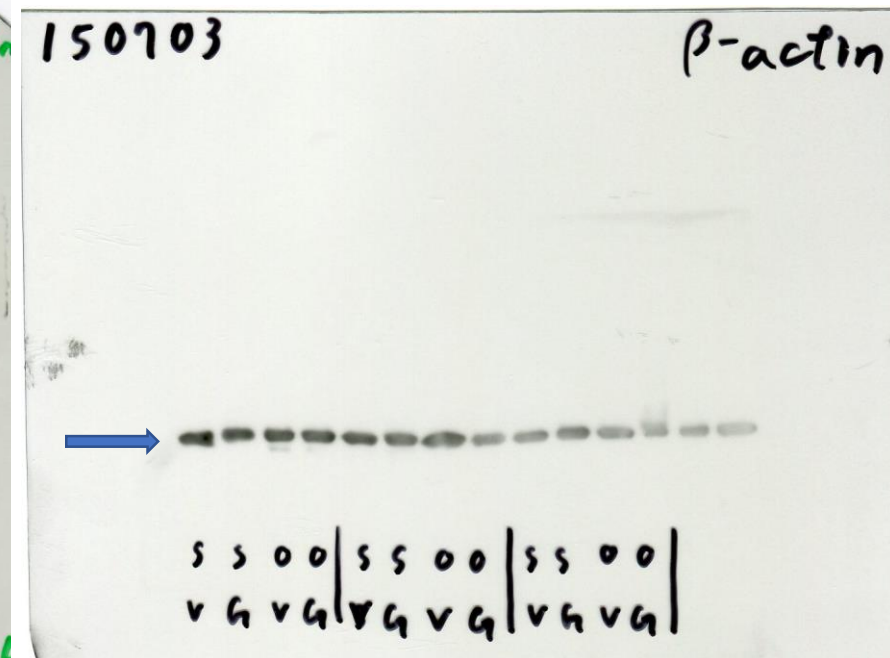

Supplement: Supplementary file 1 [file DataSheet1_v1.PDF]
