# Supplementary material for: Scabronine G Methyl Ester Improves Memory-Related Behavior and Enhances Hippocampal Cell Proliferation and Long-Term Potentiation via the BDNF-CREB Pathway in Olfactory Bulbectomized Mice
Source: Front Pharmacol. 2020 Nov 12;11:583291. doi: 10.3389/fphar.2020.583291 (PMC7689418; doi:10.3389/fphar.2020.583291)
Supplement: Supplementary file 2 [file DataSheet2_v1.PDF]

Supplemental information

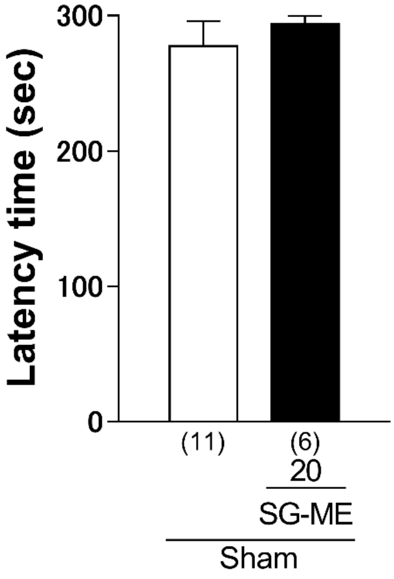

Fig. S1 Treatment with SG-ME did not affect the latency time in the passive avoidance test with sham mice. The data are expressed as the mean ± standard error of the mean. Numbers in parentheses indicate the number of animals in each group.
